# Transgenerational Sublethal Effects of Chlorantraniliprole and Emamectin Benzoate on the Development and Reproduction of *Spodoptera frugiperda*

**DOI:** 10.3390/insects14060537

**Published:** 2023-06-08

**Authors:** Xuecong Zhang, Chaoxing Hu, Lihong Wu, Wenlong Chen

**Affiliations:** Guizhou Provincial Key Laboratory for Agricultural Pest Management of the Mountainous Region, Institute of Entomology, Scientific Observing and Experimental Station of Crop Pest in Guiyang, Ministry of Agriculture, Guizhou University, Guiyang 550025, China

**Keywords:** *Spodoptera frugiperda*, chlorantraniliprole, emamectin benzoate, sublethal effects

## Abstract

**Simple Summary:**

This study documents the sublethal effects of emamectin benzoate and chlorantraniliprole on the growth, development, and reproduction of the fall armyworm (FAW), *Spodoptera frugiperda* (J. E. Smith) (Lepidoptera: Noctuidae). FAW is an invasive agricultural pest that poses a significant threat to grain production worldwide. We analyzed the age-stage, two-sex life table to understand the impact of sublethal doses (LC_10_ and LC_25_) of emamectin benzoate and chlorantraniliprole on FAW. Our findings indicate that both emamectin benzoate and chlorantraniliprole prolong the development of the FAW F_0_ generation, and fecundity was reduced at increasing concentrations of the insecticide. In the F_1_ generation, emamectin benzoate significantly reduced fecundity, and the LC_10_ dosage shortened the preadult period without affecting the adult stage. Furthermore, chlorantraniliprole lengthened the preadult and adult stages of FAW at LC_10_ and LC_25_, respectively, which significantly improved fecundity. Overall, our results indicate that emamectin benzoate effectively inhibits FAW population growth at low concentrations. In contrast, chlorantraniliprole may stimulate population growth and cause a resurgence of the FAW population.

**Abstract:**

*Spodoptera frugiperda* (J. E. Smith), commonly known as the fall armyworm (FAW), causes significant damage to many different crop species. In this study, age-stage, two-sex life table analysis was used to investigate the effects of sublethal concentrations of emamectin benzoate and chlorantraniliprole on FAW development and reproduction. In the F_0_ generation, exposure to emamectin benzoate and chlorantraniliprole significantly impacted the duration of FAW, thus prolonging the development duration of each instar, but not the prepupal stage. Furthermore, the weight of FAW pupae was significantly reduced by emamectin benzoate at LC_25_ and chlorantraniliprole at LC_25_ in the F_0_ generation. With respect to fecundity, emamectin benzoate and chlorantraniliprole significantly reduced fecundity in the F_0_ generation. In the F_1_ generation, emamectin benzoate at LC_10_ had no significant effect on the preadult or adult stages, whereas LC_25_ significantly shortened the preadult period. The preadult and adult stages of FAW exposed to chlorantraniliprole at LC_10_ and LC_25_ were significantly prolonged. Furthermore, emamectin benzoate had no significant effect on the pupal weight of the F_1_ generation. Chlorantraniliprole had no significant effect at LC_10_, but significant reduced pupal weight occurred at LC_25_ in the F_1_ generation. With respect to fecundity, emamectin benzoate significantly reduced fecundity in the F_1_ generation. Interestingly, chlorantraniliprole significantly increased fecundity in the F_1_ generation, which could promote population growth and pest resurgence. These findings have important implications for the integrated pest management of FAW and provide a reference for the more effective control of FAW.

## 1. Introduction

The fall armyworm (FAW), *Spodoptera frugiperda* (J. E. Smith) (Lepidoptera: Noctuidae), is an important pest that is native to tropical and subtropical America [1]. FAW is characterized by high reproductive capacity, strong migratory capability, and a wide host range [2,3]. Since invading China in January 2019, FAW has spread to 27 provinces and has emerged as a major agricultural pest in China [4].

Currently, FAW management relies on chemical insecticides [5,6,7]. While pesticides are generally effective in inducing strong lethal effects on targeted pests, suboptimal coverage during application and residual concentrations can result in the exposure of pests to sublethal levels of pesticides. Exposure to sublethal doses of insecticides can induce physiological and behavioral effects [8] and can impact the duration of developmental stages, life history, sex ratios, fecundity, feeding, pupation, emergence, and oviposition. There is evidence that sublethal and lethal concentrations can prolong development, reduce longevity and fecundity [9,10,11,12], and cause an increase in pest populations [13,14]. Therefore, it is critical to understand the sublethal effects of insecticides to facilitate the efficacy of pesticides and optimize pest management strategies.

In 2020, the Ministry of Agriculture and Rural Affairs (MARA) in China recommended using chlorantraniliprole and emamectin benzoate to prevent damage caused by FAW. These pesticides can effectively control FAW and other pests, while being less toxic to beneficial arthropods [15,16,17]. Chlorantraniliprole is a new generation anthranilic diamide insecticide that acts on ryanodine receptors in insects, causing disordered muscle contractions, dehydration, and the cessation of feeding on plant hosts [18,19]. When used at sublethal concentrations (LC_15_ and LC_30_), chlorantraniliprole prolonged the larval development of *Spodoptera cosmioides* and decreased adult fecundity [20]. Moreover, sublethal concentrations of chlorantraniliprole prolonged the pupal stage and oviposition period of *Plutella xylostella* and increased adult fecundity [21]. Emamectin benzoate is a macrocyclic lactone insecticide that targets neurotransmitter gamma-aminobutyric acid. This results in a continuous flow of chloride ions within muscles, leading to paralysis and the eventual death of target pests [22]. In the cabbage moth, *Mamestra brassicae*, the sublethal effects of emamectin benzoate include prolonged developmental periods for larvae and pupae and a negative effect on reproduction [23]. Studies on *Paederus fuscipes* larvae have shown that exposure to a sublethal dose (LC_30_) of emamectin benzoate can negatively influence the pre-imaginal stage and feeding potential [24]. Furthermore, the LC_30_ dose reduced fecundity, body weight, and the preoviposition period of adults from *P. fuscipes*. Further experiments with adult *P. fuscipes* revealed a significant reduction in fecundity and feeding potential at the LC_30_ dose [24]. By understanding these sublethal effects, we can better assess the impact of chlorantraniliprole and emamectin benzoate on nontarget organisms, which will lead to more effective strategies for pest management.

Research on the sublethal effects of insecticides is essential for sustainable, effective pest management. Previous studies have examined the sublethal effects of chlorantraniliprole (LC_30_) and emamectin benzoate (LC_10_ and LC_20_) on third instar FAW larvae [25,26]; however, few studies have evaluated the sublethal effects of these pesticides on earlier stage larvae. The sensitivity of lepidoptera pests to insecticides generally decreases as they mature; for example, the relative toxicity index of chlorantraniliprole for first instar larvae of *Chilo suppressalis* (Walker) is 5.63 times higher than toxicity for fourth instar larvae [27]. Furthermore, the sensitivity of *Spodoptera exigua* (Hübner) to emamectin benzoate decreased at each subsequent instar stage [28]. Therefore, the primary objective of the present study was to evaluate the lethal and sublethal effects of chlorantraniliprole and emamectin benzoate on second instar FAW using bioassays and analyses of age-stage, two-sex life tables. Our study provides a foundation for the effective application of chlorantraniliprole and emamectin benzoate, which will help efforts to control FAW outbreaks on a global scale.

## 2. Materials and Methods

### 2.1. Insects and Insecticides

The FAW population used in this study originated from Yunnan Province, China. The laboratory population was reared for over 20 generations at the Institute of Entomology, Guizhou University on an artificial diet in laboratory conditions, at 25 ± 1 °C, 75 ± 5% RH, and a photoperiod of 16:8 (L–D) h. Based on research conducted by Di et al. (2021) [29], the artificial diet formula was refined and now comprises the following components: 400 mL distilled water, 40 g soybean powder, 40 g wheat bran, 16 g yeast powder, 8 g casein, 3.2 g ascorbic acid, 8 g agar, 0.4 g choline chloride, 0.8 g sorbic acid, 0.08 g inositol, and 0.08 g cholesterol.

Emamectin benzoate (71.74% pure) and chlorantraniliprole (97.8% pure) were purchased from Guangxi Tianyuan Biochemical Co., Ltd., Nanning, China.

### 2.2. Bioassays

Bioassays were conducted with 2nd instar FAW larvae. The emamectin benzoate and chlorantraniliprole were diluted into five concentration gradients through serial dilution. The study tested five concentrations of emamectin benzoate (14 μg/g, 7 μg/g, 3.5 μg/g, 1.75 μg/g, and 0.875 μg/g), and five concentrations of chlorantraniliprole (32 μg/g, 16 μg/g, 8 μg/g, 4 μg/g, and 2 μg/g). After the artificial diet was prepared, but before it had cooled, 10 mL of insecticide was added to 100 g of the diet and thoroughly mixed. Then, 20 g of the feed containing insecticides was placed in each Petri dish, and 30 uniformly grown 2nd instar FAW larvae were added to each dish. After 48 h of exposure to the two insecticides, mortality was assessed by gently prodding the larvae with a brush; those that did not move were considered dead. Each concentration was replicated three times, and untreated larvae were used as the control group. Treated larvae were reared in controlled climate chambers at 25 ± 1 °C, 75 ± 5% RH and a photoperiod of 16 h L:8 h D.

### 2.3. Sublethal Effects of Chlorantraniliprole and Emamectin Benzoate on the Parental Generation of FAW

In this experiment, 2nd instar FAW larvae were reared on an artificial diet containing sublethal insecticide concentrations (LC_10_ and LC_25_); the control diet contained no pesticides. Larvae (*n* = 100) were used in each treatment and were transferred to separate 6-well cell culture plates for individual rearing on insecticide-free diets for 48 h post-treatment. The duration of larval development and mortality were recorded daily. The larval instars are primarily differentiated by the length of their bodies and the width of their head capsules [30,31]. Pupae were weighed and placed in individual plastic cups until adults emerged, and the duration of the pupal stage was recorded. On the first day following adult eclosion, the male and female adults were identified and mated in pairs of male and female in separate 500 mL plastic cups, which were covered with 120 mesh gauze. All adults were fed daily with 10% (*v*/*v*) honey water. The preoviposition and oviposition periods, fecundity, and longevity of adult males and females were recorded until death. FAW larvae and adults were maintained in artificial climate chambers maintained as described above.

### 2.4. Transgenerational Sublethal Effects of Chlorantraniliprole and Emamectin Benzoate on F_1_ Individuals

To assess the transgenerational effects of chlorantraniliprole and emamectin benzoate exposure on FAW offspring, 100 1st instar larvae were collected from the treatment in Section 2.3 and transferred individually to culture plates (12 wells/plate) containing pesticide-free diets. Fresh diets were provided daily, and the development time and mortality rate of FAW larvae and pupae were measured each day. Newly emerged FAW males and females were paired in 500 mL plastic cups, and the preoviposition and oviposition periods, fecundity, and longevity of adult males and females were recorded daily until death.

### 2.5. Life Table Study

Raw data for the life table were recorded based on the age-stage, two-sex life table theory [32,33]. Basic life table parameters, including the adult preoviposition period (APOP), total preoviposition period (TPOP), oviposition period (OP), age-stage-specific survival rates (*s_xj_*), female age-stage-specific fecundity (*f_xj_*), population age-specific survival rates (*l_x_*), population age-specific fecundity (*m_x_*), age-specific life expectancy (*e_xj_*), and age-stage-specific reproductive values (*v_xj_*), were calculated using the computer program TWOSEX-MSChart [34,35]. The curves for *s_xj_*, *f_xj_*, *l_x_*, *m_x_*, *l_x_m_x_*, *e_xj_*, and *v_xj_* were plotted for each treatment using SigmaPlot.

Parameters of the FAW population were calculated, including the net reproductive rate *R*_0_ (the number of offspring produced by an individual during its lifetime) and the intrinsic rate of increase *r* (the rate of population increase per unit of time). Other measured parameters included the finite rate of increase *λ* (the multiple of the population’s daily growth under the condition of unlimited resources) and the mean generation time *T* (the time it takes to increase *R_0_* when a population reaches a steady growth rate).

$$R_{0}=\overset{\infty }{\underset{x=0}{\sum }}l_{x}m_{x}\;$$

$$\overset{\infty }{\underset{x=0}{\sum }}e^{-r\left(x+1\right)}l_{x}m_{x}=1$$

$$\lambda =e^{r}$$

$$T=\frac{\ln R_{0}}{r}$$

### 2.6. Data Analysis

To calculate the sublethal concentrations (LC_10_ and LC_25_), mortality data from the larval toxicity experiment were subjected to probit regression analysis against the log insecticide concentration using SPSS v. 23.0 (IBM Corp., Armonk, NY, USA).

TWOSEX-MSChart 2022 (http://140.120.197.173/Ecology/prod02.htm, accessed on 4 March 2023) was used to calculate the life table parameters. The life table parameters for sublethal concentrations of chlorantraniliprole and emamectin benzoate were calculated using the bootstrap method with 100,000 resampled data points for estimating the means and standard errors (SE) [36]. Differences between population data, development time, and reproductive values were estimated using the paired bootstrap test in the TWOSEX-MSChart (*p* < 0.05). Sigmaplot v. 12.5 software was used to plot the figures.

## 3. Results

### 3.1. LC Values for Chlorantraniliprole and Emamectin Benzoate

Bioassay results for second instar larvae are shown in Table 1. The LC_10_, LC_25_, and LC_50_ values for chlorantraniliprole were 1.725, 3.921, and 9.763 μg/g, respectively, whereas values for emamectin benzoate were 3.585, 5.162, and 7.739 μg/g, respectively.

### 3.2. Effects of Sublethal Concentrations of Emamectin Benzoate and Chlorantraniliprole on Development and Pupal Weight in the F_0_ Generation

Table 2 shows the impact of sublethal concentrations of emamectin benzoate and chlorantraniliprole on the duration of the development and pupal weight of the FAW F_0_ generation. Sublethal dosages of the two pesticides prolonged the transition from second to fifth instar, with a greater effect at LC_25_ compared to LC_10_. There were no significant changes for the duration of the pupal and adult stages. Pupal weight was significantly lower at the LC_25_ of emamectin benzoate and chlorantraniliprole compared to the untreated control.

### 3.3. Effects of Sublethal Concentrations of Emamectin Benzoate and Chlorantraniliprole on Adult Longevity and Fecundity in the F_0_ Generation

Sublethal concentrations (LC_10_ and LC_25_) of emamectin benzoate and chlorantraniliprole did not alter the longevity of FAW adults from the F_0_ generation; however, sublethal dosages did significantly lengthen the adult preoviposition period (APOP) (Table 3). Exposure to sublethal dosages of emamectin benzoate and chlorantraniliprole resulted in a significant decrease in fecundity compared to the control group. The reduction in fecundity was more pronounced at higher concentrations of emamectin benzoate and chlorantraniliprole.

### 3.4. Effects of Sublethal Concentrations of Emamectin Benzoate and Chlorantraniliprole on Life Table Parameters

The effect of sublethal concentrations of emamectin benzoate and chlorantraniliprole on the population growth of the F_0_ generation was investigated (Table 4). Sublethal doses of the two chemicals had a negative impact on FAW life table parameters, with *r*, *λ*, and *R_0_* decreasing as the pesticide concentration increased. The *r* parameter of the LC_10_ and LC_25_ of emamectin benzoate was reduced by 7.3% and 19.6% relative to the control, respectively, whereas the LC_10_ and LC_25_ of chlorantraniliprole was 16.1% and 39.7% lower than the control, respectively. Furthermore, the *R_0_* parameter of the LC_10_ and LC_25_ of the chlorantraniliprole and emamectin benzoate groups saw a considerable decline of 40% and 75.6% and 12.9% and 39.7%, respectively.

### 3.5. Effects of Sublethal Concentrations of Emamectin Benzoate and Chlorantraniliprole on Development and Pupal Weight in the F_1_ generation

The use of emamectin benzoate at LC_10_ increased the development time for eggs, fourth–sixth instar larvae, prepupa, and preadult stages compared to the control group (Table 5). In contrast, the first, second, and third instar larvae, pupae, and adults exhibited significantly shorter developmental periods compared to the untreated control. At the LC_25_ dose of emamectin benzoate, the duration of the fourth instar larval stage was significantly longer than the control group, but there was no significant difference in the pupal weight. When chlorantraniliprole was administered at LC_10_, eggs, fifth and sixth instar larvae and prepupal, preadult, and adult stages showed significant lengthening compared to the control group. At LC_25_, eggs, fourth–sixth stage instars, and prepupal, preadult, and adult stages were significantly longer than the control group; in contrast, the pupal stage was significantly shortened and pupal weight was significantly decreased. In summary, emamectin benzoate at LC_10_ caused a significant increase in development time for several different growth stages, while the use of chlorantraniliprole at LC_10_ and LC_25_ prolonged development for several stages and decreased pupal weight.

### 3.6. Effects of Emamectin Benzoate and Chlorantraniliprole on Adult Longevity and Fecundity in the F_1_ Generation

The effects of emamectin benzoate and chlorantraniliprole on adult longevity, APOP, TPOP, and fecundity were analyzed (Table 6). Emamectin benzoate did not alter adult longevity or the APOP; however, the LC_10_ dose shortened TPOP, and LC_25_ prolonged TPOP. Moreover, the fecundity of FAW at LC_10_ (1146.25 ± 81.87) and LC_25_ (1201.63 ± 136.06) exhibited a significant reduction in comparison to the control group (CK) (1357.23 ± 140.13). Chlorantraniliprole at LC_10_ had a greater impact on adult longevity, APOP, and TPOP than the LC_25_ dose. When administered at LC_10_, chlorantraniliprole significantly increased adult longevity, APOP, and TPOP; however, the LC_25_ dosage of chlorantraniliprole prolonged TPOP but did not significantly alter adult longevity or APOP. Interestingly, the fecundity of (FAW) at LC_10_ (1408.12 ± 154.64) and LC_25_ (1669.40 ± 199.25) showed a significant improvement compared to the control (CK) group (1357.23 ± 140.13).

### 3.7. Effects of Sublethal Emamectin Benzoate and Chlorantraniliprole on FAW Survival

**Figure 1:**
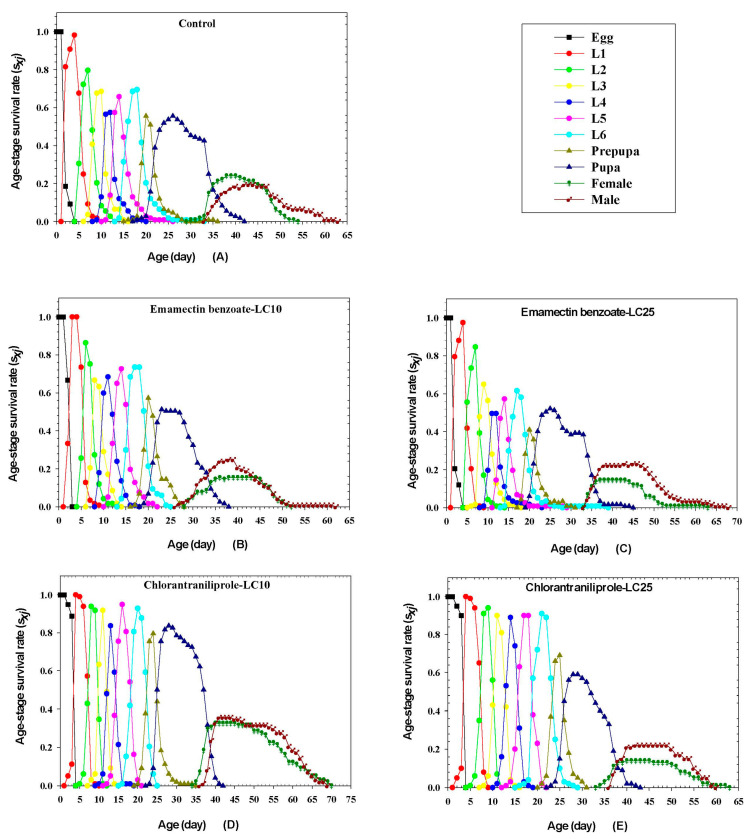
Effects of sublethal emamectin benzoate and chlorantraniliprole on age-stage-specific survival rates (*s_xj_*) of FAW in the F_1_ generation. Abbreviations: L1–L6 indicate 1st to 6th instar larvae.

The age-stage-specific survival rates (*s_xj_*) of FAW exposed to sublethal doses of emamectin benzoate and chlorantraniliprole are shown in Figure 1. The survival rate of the eggs developmental stage over 90% for both insecticides and the untreated control. Males on sublethal doses of emamectin benzoate exhibited lower survival rates (LC_10_, 15.38%; LC_25_, 14.53%) compared to the control (24.07%). In contrast, exposure of larvae to sublethal doses of chlorantraniliprole resulted in increased survival rates; this was very obvious for survival rates at the LC_10_ dose (females, 35.71%; males, 32.65%), which was significantly higher than the untreated control group (females, 19.444%; males, 24.07%).

### 3.8. Effects of Sublethal Emamectin Benzoate and Chlorantraniliprole on FAW Fecundity

**Figure 2:**
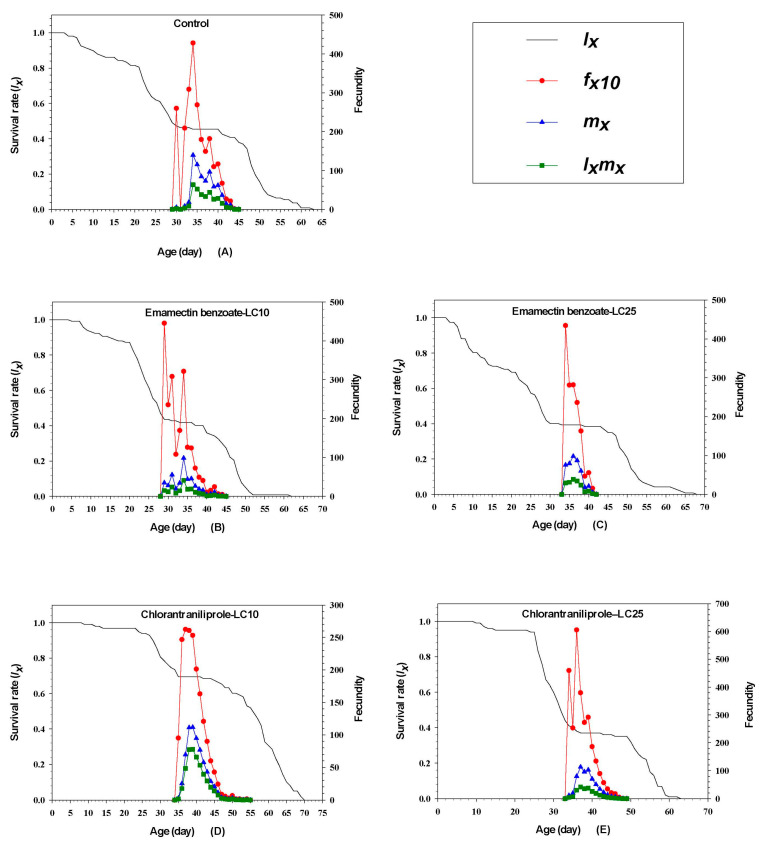
Effects of sublethal concentrations of emamectin benzoate and chlorantraniliprole on age-stage-specific survival rates (*l_x_*), female age-specific fecundity (*f_x10_*), age-specific fecundity of the total population (*m_x_*) and age-specific maternity (*l_x_m_x_*) of FAW in the F_1_ generation.

Figure 2 shows the daily fecundity (number of eggs/day) of female FAW at age 10 (*f_x_*). The *l_x_* parameter represents age-stage-specific survival rates, *m_x_* shows age-specific fecundity of the total population, and the *l_x_m_x_* parameter represents age-specific net maternity. The *f_x10_*, *m_x_*, and *l_x_m_x_* increased for FAW treated with chlorantraniliprole and the control group before decreasing, and the reproductive curves (*f_x10_*, *m_x_*, and *l_x_m_x_*) started on the 31st, 36th, and 35th, respectively. The *m_x_* and *l_x_m_x_* increased for FAW treated with emamectin benzoate before decreasing, and the *f_x10_* of emamectin benzoate decreased, and the reproductive curves (*f_x10_*, *mx*, and *l_x_m_x_*) started on the 30th and 35th, respectively.

### 3.9. Effects of Sublethal Chlorantraniliprole and Emamectin Benzoate on FAW Life Expectancy

**Figure 3:**
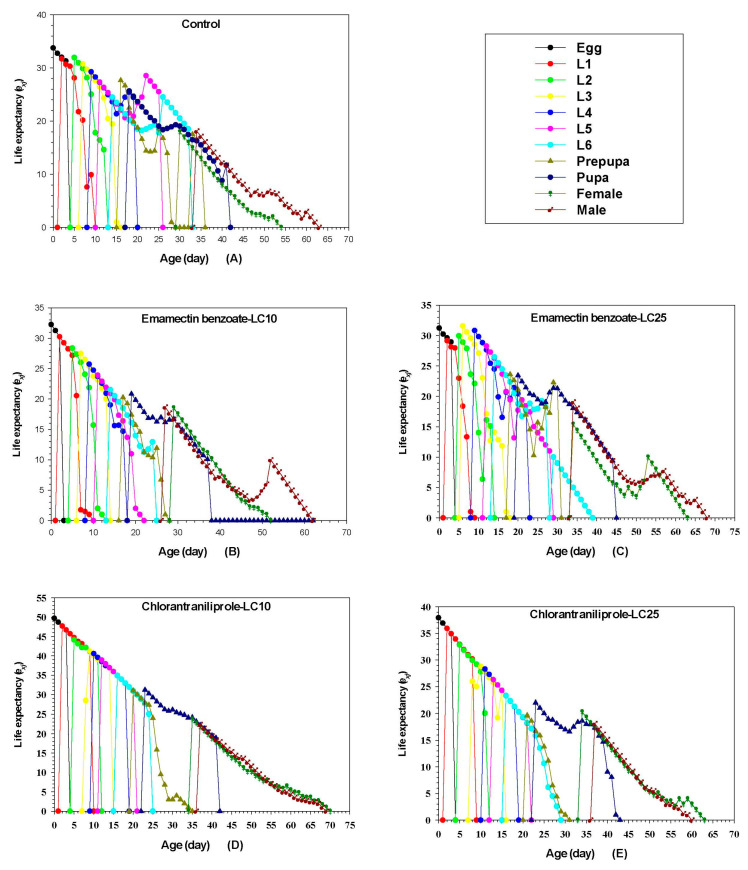
Effect of sublethal concentrations of emamectin benzoate and chlorantraniliprole on age-stage-specific life expectancy (*e_xj_*) of the FAW F_1_ generation.

The *e_xj_* represents the time that an individual of age *x* and stage *j* is expected to live after age *x* (Figure 3). The *e_xj_* of all individuals decreased as the age of larval instars increased. The average longevity of the control group was 33.75 d, which was higher than the mean longevity of sublethal doses of emamectin benzoate (LC_10_, 32.25 d; LC_25_, 31.23 d). Longevity was highest for sublethal doses of chlorantraniliprole (LC_10_, 49.72 d; LC_25_, 37.94 d).

### 3.10. Effects of Sublethal Chlorantraniliprole and Emamectin Benzoate on FAW Reproduction

**Figure 4:**
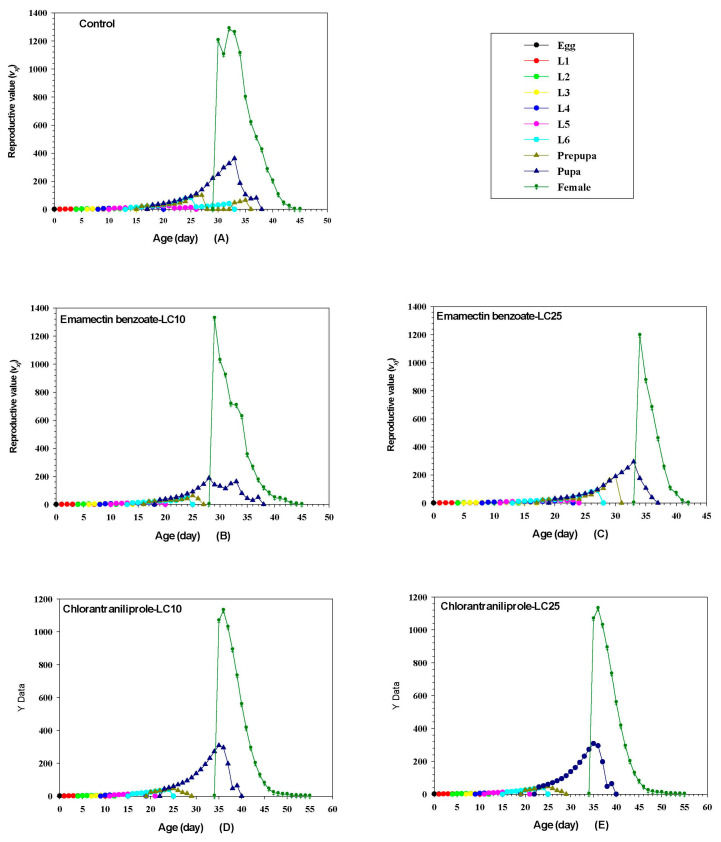
Effect of sublethal concentrations of emamectin benzoate and chlorantraniliprole on age-specific reproductive values (*v_xj_*) of FAW in the F_1_ generation.

The *v_xj_* of FAW represents the contribution of all individuals at age *x* and stage *j* to reproduction (Figure 4). The initial reproductive value for the control group was 1.17, and pesticide-treated groups had lower reproductive values than the control group (chlorantraniliprole: LC_10_, 1.16; LC_25_, 1.15; emamectin benzoate: LC_10_, 1.16; LC_25_, 1.15). The *v_xj_* curve showed an upward trend with the increase of age and developmental stage. Female adults in the control group reached their highest reproductive values on the 33rd, with a value of 1287.59. Female adults treated with LC_10_ and LC_25_ doses of emamectin benzoate reached their highest reproductive values at 30th and 35th d with values of 1327.09 and 1194.97, respectively. Female adults exposed to the LC_10_ and LC_25_ doses of chlorantraniliprole reached peak reproductive values at 35th and 37th d with values of 1885.42 and 1130.4, respectively.

### 3.11. Effects of Sublethal Emamectin Benzoate and Chlorantraniliprole on FAW Life Table Parameters

Table 7 illustrates the impact of sublethal concentrations of emamectin benzoate and chlorantraniliprole on life table parameters for the FAW F_1_ population. Exposure to both emamectin benzoate and chlorantraniliprole resulted in significant decreases in the intrinsic rate of increase (*r*) and finite rate of increase (*λ*), and inhibition increased according to the concentration. Interestingly, the LC_10_ of chlorantraniliprole increased the net reproductive rate (*R_0_*); however, all other insecticide concentrations exhibited a significant reduction in *R_0_*. Additionally, chlorantraniliprole significantly increased the mean generation time (*T*), while emamectin benzoate significantly reduced *T*.

### 3.12. Effects of Sublethal Emamectin Benzoate and Chlorantraniliprole on Projected FAW Populations

**Figure 5:**
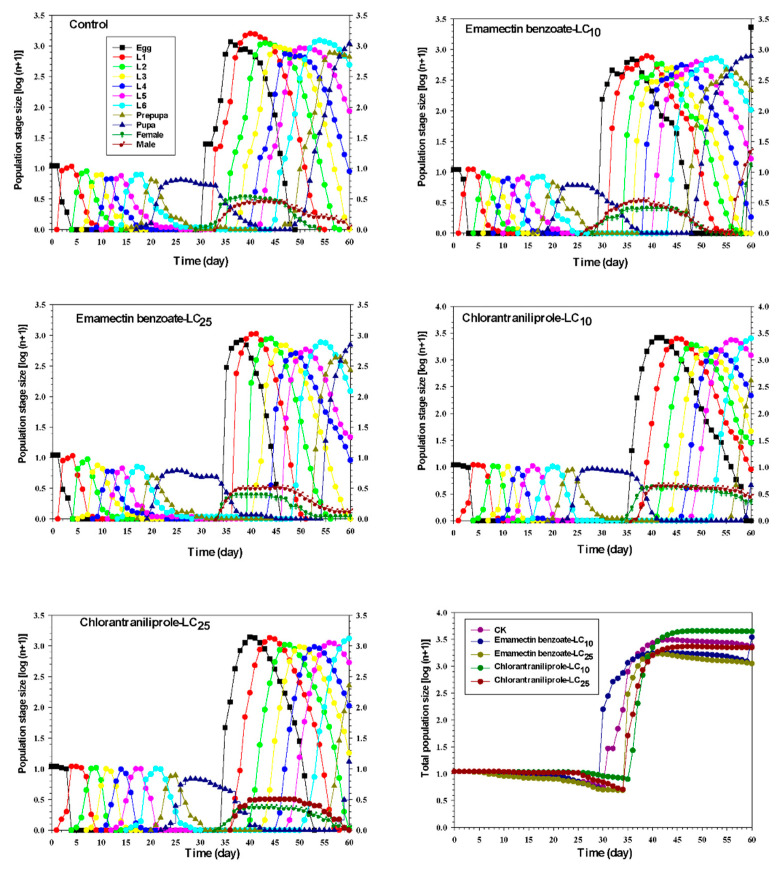
Population projections for FAW (F_1_ generation) exposed to sublethal concentrations of emamectin benzoate and chlorantraniliprole.

Projected population growth for different FAW developmental stages and groups after exposure to sublethal concentrations of emamectin benzoate and chlorantraniliprole is shown in Figure 5. Estimated population growth in response to the LC_10_ dose of emamectin benzoate (3.54) and chlorantraniliprole (3.65) was higher than the control (3.37), whereas the LC_25_ estimates of emamectin benzoate (3.05) and chlorantraniliprole (3.35) were lower than the control (3.37).

## 4. Discussion

Chlorantraniliprole and emamectin benzoate are highly effective, broad-spectrum insecticides used to control lepidopteran pests such as *Helicoverpa armigera*, *P. xylostella*, and *S. frugiperda*. In this study, the median lethal concentrations (LC_50_) of chlorantraniliprole and emamectin benzoate for FAW were 9.763 and 7.739 μg/g, respectively; these concentrations are 1.61- and 3.43-fold lower than the LC_50_ reported elsewhere [37]. This difference could be attributed to the utilization of second instar larvae in the present investigation, whereas third instar larvae were employed in the previous study [37]. It was reported that the sensitivity of lepidoptera pests to insecticides generally decreases as they mature; for example, the relative toxicity index of chlorantraniliprole for first instar larvae of *Chilo suppressalis* (Walker) is 5.63 times higher than the toxicity for fourth instar larvae [27]. Our study explores the impact of sublethal concentrations of emamectin benzoate and chlorantraniliprole insecticides on FAW population dynamics across each stage of growth and development. Our findings indicate that exposure to sublethal concentrations of these insecticides has variable effects on the duration of developmental stages in the F_0_ generation and except prepupa stage.. Interestingly, the second to fifth instar larval stages were significantly prolonged. In the F_1_ generation, the preadult period was significantly reduced by emamectin benzoate at LC_10_, which could be mainly attributed to shortening of the pupa period; however, there was no significant change in the duration of the adult stage. Emamectin benzoate at LC_25_ did not affect the total development time of the F_1_ generation. When FAW was exposed to chlorantraniliprole at LC_10_ and LC_25_, the preadult stage was prolonged by 2.86 and 2.3 d, respectively, and the adult stage was prolonged by 6.37 and 2.59 d compared to the control, respectively. Our results also show that the pupal weight of FAW was reduced by the two insecticides in both the F_0_ and F_1_ generations. Emamectin benzoate at the LC_25_ dose resulted in a significant difference in the F_0_ but not the F_1_ generation. Although no significant differences were observed with chlorantraniliprole at LC_10_, a remarkable variation was observed at the LC_25_ dose. These results emphasize the effects of sublethal concentrations of insecticides on pest population dynamics and suggest the need for further research.

A related study also reported that the development of *Spodoptera litura* was prolonged with sublethal concentrations of chlorantraniliprole [38]. Chlorantraniliprole at LC_15_ and LC_30_ increased the larval stage by 174.23% and 125.62%, respectively, and significantly prolonged the adult stage of *S. cosmioides* [20]. Another study showed that chlorantraniliprole could adversely impact the transition from larvae to pupae in silkworms, potentially by targeting the gene encoding *Ftz-f1* [39]. Chlorantraniliprole may also perturb the normal development of neuronal tissue, which might explain why development duration were altered [9]. Similarly, the sublethal effects of emamectin benzoate have been confirmed in other insects; for example, the sublethal dose of emamectin benzoate delayed *H armigera* growth and development and prolonged pupation [40]. Gao et al. reported that sublethal doses of emamectin benzoate inhibited the feeding and growth of *Bombyx mori* larvae [41]. Sublethal concentrations of emamectin benzoate decreased the developmental duration of larvae and reduced pupation in *S. exigua* [42]. It is noteworthy that emamectin benzoate induces changes in the permeability of cellular membranes to chloride ions by activating ion channels; this results in a large influx of chloride ions, loss of normal physiological functions, disturbance of nerve conduction, and parasite numbness and ulcers [22]. Moreover, exposure to emamectin benzoate induced apoptosis in FAW Sf-9 cells [43]. Further experiments are needed to clarify the mode of action for emamectin benzoate in controlling FAW.

Assessing the impact of insecticides on pest reproduction, particularly at a multiple generational level, can reveal the potential correlation between insecticide use and pest resurgence [44,45,46]. In the present study, exposure to emamectin benzoate significantly decreased the fecundity of the F_0_ and F_1_ generations. Exposure of the F_0_ generation to the LC_10_ and LC_25_ of emamectin benzoate lowered fecundity by approximately 390 and 607 eggs, respectively, and the reduction was significantly lower than the control (1252 eggs). Moreover, exposure to emamectin benzoate significantly reduced the number of eggs laid by the F_1_ generation by about 211 and 156 eggs, respectively, which was significantly lower than the control. Similarly, a significant decrease in egg production was reported when *Acartia clausi* adults received sublethal doses of emamectin benzoate, and reduced fecundity was greater at higher concentrations [47]. Similar results were reported for *S. exigua* exposed to low concentrations of emamectin benzoate [42]. Furthermore, reduced fecundity in FAW was associated with a downregulated expression of the vitellogenin gene [48]. Collectively, these findings support our results and show that emamectin benzoate inhibits pest reproduction at low concentrations. The sublethal effects of emamectin benzoate on the biological and fecundity of FAW provide valuable insights for optimizing integrated pest management strategies.

Our results show that sublethal doses of chlorantraniliprole impact fecundity in both the F_0_ and F_1_ generations. Exposure to chlorantraniliprole at LC_10_ and LC_25_ in the FAW F_0_ generation significantly decreased fecundity by 43.05% and 59.82%, respectively. In contrast, exposure to chlorantraniliprole increased fecundity in the FAW F_1_ generation, with oviposition increasing at the LC_10_ and LC_25_ dosages by 3.75% and 23%, respectively. Interestingly, Wu et al. reported different results, with fecundity in the F_0_ and F_1_ generations of FAW decreasing by 67.33% and 27.99%, respectively, after the exposure of the third FAW instar to chlorantraniliprole at LC_30_ [25]; this discrepancy may be attributable to the use of different FAW strains, instars, or sublethal doses. Exposure to different sublethal doses of dinotefuran (LC_10_, L_20_, and LC_30_) significantly reduced egg production in the F_0_ generation of *Rhopalosiphum padi*; however, exposure to lower doses (LC_10_ and LC_20_) in the F_1_ generation increased fecundity [49]. Furthermore, when the sublethal concentration increased to LC_30_, fecundity in the F_1_ generation was inhibited [49]. These results indicate that lower dosages of dinotefuran can stimulate growth and reproduction in the F_1_ generation, thereby leading to pest resurgence. Similarly, Yin et al. reported that sublethal concentrations of chlorantraniliprole stimulated fecundity in *P. xylostella*, which increased by 10.28% and 28.02% at the LC_25_ and LC_50_ doses, respectively [21]. Collectively, these findings indicate that low levels of select pesticides may stimulate fecundity and increase the resurgence of insect pests.

The analysis of FAW life table parameters revealed that sublethal doses of emamectin benzoate (LC_10_ and LC_25_) caused a significant decrease in *R_0_*, *r*, and *λ* in the F_0_ generation of FAW. Furthermore, when the F_0_ generation of FAW was exposed to sublethal doses of emamectin benzoate, the F_1_ generation exhibited transgenerational effects that led to significant reductions in *R_0_*, *T*, *r*, and *λ*. Similarly, when *H armigera* was exposed to sublethal doses of carbamoyl, the *r*, *λ*, and *R_0_* were significantly reduced, whereas *T* and the population doubling time were extended [40]. Previous studies also suggested a reduction in the *r*, *λ*, and *R_0_* of *S. litura*, *Chrysoperla carnea*, and *Trichogramma brassicae* after exposure to emamectin benzoate [50,51,52]. In summary, these findings suggest that emamectin benzoate can interfere with pest population density, thereby hindering the re-establishment of the population. In contrast, exposure of the FAW F_0_ population to sublethal chlorantraniliprole resulted in a significant reduction in the *r*, *λ*, and *R_0_* relative to the control group. However, when the F_0_ generation of FAW was exposed to the LC_10_ dose of chlorantraniliprole, the *r* and *λ* for the F_1_ generation were reduced, *T* was prolonged, and *R_0_* increased, indicating that parental exposure to chlorantraniliprole at LC_10_ could stimulate the growth of the offspring population and cause a potential resurgence of FAW.

This study suggests that chlorantraniliprole and emamectin benzoate exhibit strong efficacy as pesticides against FAW populations as they have been observed to significantly inhibit both the growth and reproduction of FAW in the F_0_ generation even when administered in sublethal doses. However, in the F_1_ generation, chlorantraniliprole may stimulate the fecundity of FAW, which could potentially result in a resurgence of this pest. Therefore, it is crucial use chlorantraniliprole with caution in practical applications. To prevent sublethal effects, it is necessary to ensure a sufficient concentration or dosage of chlorantraniliprole based on the developmental stage of FAW in the field. Other factors must also be taken into account, such as the frequency of pesticide spraying, the method of insecticide application, the resistance of FAW to chlorantraniliprole, as well as the weather conditions before and after spraying. To comprehensively control FAW, chlorantraniliprole can be used in combination with other insecticides that target Lepidoptera or with other biological and agricultural control measures.

## 5. Conclusions

In this study, we utilized the commonly used doses of LC_10_ and LC_25_ to examine the sublethal impacts of emamectin benzoate and chlorantraniliprole on FAW. Emamectin benzoate is an effective insecticide for suppressing FAW population growth at low concentrations. Sublethal concentrations of emamectin benzoate significantly reduced the fecundity of both the F_0_ and F_1_ generations, which was evident by the reduction in the *R_0_*, *r*, and *λ* parameters compared to the control group. In contrast, chlorantraniliprole inhibited the growth, development, and reproduction of FAW in the F_0_ generation but promoted the growth of the F_1_ generation, which may lead to a resurgence of the FAW population. Thus, it is important to consider the potential risk of FAW population growth when using chlorantraniliprole. More research is clearly needed to fully understand the efficacy and drawbacks of using these insecticides in the field.

## Figures and Tables

**Table 1:** Toxicity of chlorantraniliprole and emamectin benzoate for 2nd instar larvae of FAW.

Insecticides	LC_10_ (μg/g) (95% CL)	LC_25_ (μg/g) (95% CL)	LC_50_ (μg/g) (95% CL)	Regression Equation	*χ* ^2^	*df*
Chlorantraniliprole	1.725 (0.866–2.626)	3.921 (2.562–5.233)	9.763 (7.605–12.662)	Y = −1.685 + 1.703X	0.430	3
Emamectin benzoate	3.585 (1.718–4.886)	5.162 (3.238–6.489)	7.739 (6.060–9.610)	Y = −3.408 + 3.835X	1.191	3

LC, CL, and *χ*^2^ indicate lethal concentration, confidence limit, and chi-square, respectively.

**Table 2:** Effects of sublethal concentrations of emamectin benzoate and chlorantraniliprole on development duration and pupal weight of FAW in the F_0_ generation.

Developmental Stages	Developmental Duration (d)				
	CK	Emamectin Benzoate		Chlorantraniliprole	
		LC_10_	LC_25_	LC_10_	LC_25_
2nd instar larva (d)	2.08 ± 0.04 d	2.38 ± 0.07 c	2.65 ± 0.08 b	2.63 ± 0.09 b	3.23 ± 0.11 a
3th instar larva (d)	2.13 ± 0.04 c	2.66 ± 0.17 a	2.47 ± 0.14 ab	2.40 ± 0.10 ab	2.68 ± 0.11 a
4th instar larva (d)	2.16 ± 0.04 c	2.98 ± 0.15 a	2.83 ± 0.13 ab	2.54 ± 0.11 b	3.02 ± 0.14 a
5th instar larva (d)	2.68 ± 0.05 b	3.33 ± 0.19 a	3.34 ± 0.12 a	3.25 ± 0.10 a	3.48 ± 0.15 a
6th instar larva (d)	4.03 ± 0.07 b	4.53 ± 0.22 a	4.20 ± 0.11 ab	4.30 ± 0.12 ab	4.48 ± 0.22 a
Prepupa (d)	2.27 ± 0.07 a	2.13 ± 0.08 ab	2.02 ± 0.06 b	2.30 ± 0.07 a	2.24 ± 0.09 a
Pupa (d)	12.80 ± 0.25 a	13.37 ± 0.28 a	13.00 ± 0.18 a	13.43 ± 0.21 a	13.56 ± 0.27 a
Adult (d)	13.16 ± 1.21 a	13.46 ± 0.65 a	14.61 ± 0.60 a	15.07 ± 1.08 a	14.25 ± 1.52 a
Pupal weight (g)	0.2270 ± 0.03 a	0.2174 ± 0.04 ab	0.2137 ± 0.03 b	0.2151 ± 0.03 ab	0.2088 ± 0.04 b

Data are means ± SE. Values in the same row followed by different letters are significantly different when analyzed by the paired bootstrap test (*p* < 0.05). Abbreviations: CK, untreated control.

**Table 3:** Effects of emamectin benzoate and chlorantraniliprole on fecundity and longevity of FAW adults in the F_0_ generation.

Parameters			Emamectin Benzoate		Chlorantraniliprole	
	Gender	CK	LC_10_	LC_25_	LC_10_	LC_25_
Adult longevity (d)	Male	12.80 ± 1.89 a	13.47 ± 0.78 a	14.16 ± 0.61 a	13.56 ± 1.14 a	13.20 ± 2.00 a
	Female	13.70 ± 1.22 a	13.45 ± 1.19 a	15.63 ± 1.42 a	17.80 ± 2.02 a	16.00 ± 2.34 a
Preoviposition (d) (APOP)	Female	3.90 ± 0.38 b	5.45 ± 0.43 a	5.91 ± 0.49 a	5.40 ± 0.50 a	5.83 ± 0.75 a
Fecundity (eggs/female)	Female	1252.36 ± 174.59 a	861.74 ± 133.77 b	645.95 ± 139.34 d	713.27 ± 243.25 c	503.23 ± 191.54 e

Data are means ± SE. Values in the same row followed by different letters are significantly different when analyzed by the paired bootstrap test (*p* < 0.05). Abbreviations: APOP, adult preoviposition period; CK, untreated control.

**Table 4:** Population parameters of the FAW F_0_ generation in response to sublethal doses of emamectin benzoate and chlorantraniliprole.

Parameters	CK	Emamectin Benzoate		Chlorantraniliprole	
		LC_10_	LC_25_	LC_10_	LC_25_
Intrinsic rate of increase (*r*/day)	0.1635 ± 0.01 a	0.1516 ± 0.01 b	0.1314 ± 0.01 d	0.1372 ± 0.02 c	0.1045 ± 0.02 e
Finite rate of increase (*λ*/day)	1.1777 ± 0.01 a	1.1637 ± 0.01 b	1.1405 ± 0.01 d	1.1472 ± 0.02 c	1.1104 ± 0.03 e
Net reproductive rate (*R_0_*/offspring per individual)	126.50 ± 41.54 a	110.17 ± 34.99 b	76.32 ± 26.67 c	75.90 ± 33.53 d	30.92 ± 16.09 e

Data are means ± SE. Values in the same row followed by different letters are significantly different when analyzed by the paired bootstrap test (*p* < 0.05). CK, untreated control.

**Table 5:** Effects of emamectin benzoate and chlorantraniliprole on development and pupal weight in the FAW F_1_ generation.

Developmental Stages	Developmental Duration (d)				
		Emamectin Benzoate		Chlorantraniliprole	
	CK	LC_10_	LC_25_	LC_10_	LC_25_
Egg (d)	2.28 ± 0.06 c	2.67 ± 0.04 b	2.32 ± 0.06 c	3.84 ± 0.05 a	3.85 ± 0.05 a
1st instar larva (d)	3.66 ± 0.07 a	3.14 ± 0.08 b	3.16 ± 0.04 b	3.70 ± 0.06 a	3.81 ± 0.06 a
2nd instar larva (d)	2.90 ± 0.07 a	2.3 ± 0.06 c	3.05 ± 0.07 a	2.71 ± 0.06 b	2.90 ± 0.04 a
3th instar larva (d)	2.62 ± 0.07 a	2.25 ± 0.05 b	2.72 ± 0.09 a	2.25 ± 0.04 b	2.76 ± 0.05 a
4th instar larva (d)	2.11 ± 0.06 c	2.66 ± 0.08 a	2.36 ± 0.10 b	2.27 ± 0.05 bc	2.83 ± 0.06 a
5th instar larva (d)	2.91 ± 0.11 cd	3.04 ± 0.05 c	2.77 ± 0.08 d	3.79 ± 0.06 a	3.47 ± 0.06 b
6th instar larva (d)	3.93 ± 0.07 c	3.97 ± 0.06 c	4.04 ± 0.06 bc	4.18 ± 0.04 ab	4.36 ± 0.08 a
Prepupa (d)	2.07 ± 0.05 c	2.07 ± 0.09 c	2.09 ± 0.06 c	2.69 ± 0.06 a	2.32 ± 0.06 b
Pupa (d)	12.98 ± 0.20 a	10.22 ± 0.32 c	13.47 ± 0.21 a	12.90 ± 0.14 a	11.83 ± 0.20 b
Preadult (d)	35.48 ± 0.33 b	32.37 ± 0.40 c	35.73 ± 0.32 b	38.34 ± 0.18 a	37.78 ± 0.28 a
Adult (d)	14.44 ± 0.64 c	14.06 ± 0.60 c	15.96 ± 0.81 bc	20.81 ± 0.81 a	17.03 ± 0.56 b
Pupal weight (g)	0.2275 ± 0.03 a	0.2271 ± 0.04 a	0.2184 ± 0.03 ab	0.2176 ± 0.03 ab	0.2094 ± 0.04 b

Data are means ± SE. Values in the same row followed by different letters are significantly different when analyzed by the paired bootstrap test (*p* < 0.05).

**Table 6:** Effects of emamectin benzoate and chlorantraniliprole on adult longevity and reproduction in the FAW F_1_ generation.

Parameters			Emamectin Benzoate		Chlorantraniliprole	
	Gender	CK	LC_10_	LC_25_	LC_10_	LC_25_
Adult longevity (d)	Male	15.55 ± 1.21 bc	13.37 ± 0.76 c	16.79 ± 1.10 b	20.19 ± 1.13 a	16.68 ± 0.69 b
	Female	14.47 ± 1.81 b	15.16 ± 0.93 b	14.59 ± 1.08 b	21.50 ± 1.15 a	17.57 ± 0.94 b
APOP (d)	Female	5.80 ± 0.62 b	5.68 ± 0.36 b	5.39 ± 0.45 b	8.79 ± 0.86 a	7.64 ± 0.96 ab
TPOP (d)	Female	34.50 ± 0.32 d	32.42 ± 0.23 e	34.82 ± 0.23 c	37.31 ± 0.21 a	36.71 ± 0.40 b
Mean fecundity (eggs/female)	Female	1357.23 ± 140.13 c	1146.25 ± 81.87 d	1201.63 ± 136.06 e	1408.12 ± 154.64 b	1669.40 ± 199.25 a

Data are means ± SE. Values in the same row followed by different letters were significantly different when analyzed by the paired bootstrap test (*p* < 0.05). Abbreviations: APOP, adult preoviposition period; CK, untreated control; TPOP, total preoviposition period.

**Table 7:** Life table parameters of the FAW F_1_ generation after exposure to sublethal doses of emamectin benzoate and chlorantraniliprole.

Parameters	CK	Emamectin Benzoate		Chlorantraniliprole	
		LC_10_	LC_25_	LC_10_	LC_25_
Intrinsic rate of increase (*r*)	0.1551 ± 0.01 a	0.1517 ± 0.01 b	0.1382 ± 0.01 d	0.1506 ± 0.01 c	0.1377 ± 0.01 e
Finite rate of increase (*λ*)	1.1678 ± 0.01 a	1.1639 ± 0.01 b	1.1482 ± 0.01 d	1.1625 ± 0.01 c	1.1476 ± 0.01 e
Net reproductive rate (*R_0_*)	327.07 ± 64.87 b	186.19 ± 41.32 d	174.59 ± 43.72 e	459.93 ± 83.84 a	234.06 ± 63.77 c
Mean generation time (*T*)	37.21 ± 0.35 c	34.29 ± 0.63 e	37.12 ± 0.26 d	40.61 ± 0.32 a	39.34 ± 0.43 b

Data are means ± SE. Values in the same row followed by different letters are significantly different when analyzed by the paired bootstrap test (*p* < 0.05).

## Data Availability

Dataset is available from the first author on request.
